# The Burden of Inpatient Hospitalizations with Cardiac and Cerebrovascular Diseases in Patients with Type 1 Diabetes: Insights from the National Inpatient Sample in the US

**DOI:** 10.3390/diagnostics14151607

**Published:** 2024-07-25

**Authors:** Chun Shing Kwok, Adnan I. Qureshi, Anne Phillips, Gregory Y. H. Lip, Wasim Hanif, Josip Andelo Borovac

**Affiliations:** 1 Department for Post-Qualifying Healthcare Practice, Birmingham City University, Birmingham B15 3TN, UK; shingkwok@doctors.org.uk (C.S.K.); anne.phillips@bcu.ac.uk (A.P.); hanif.wasim@uhb.nhs.uk (W.H.); 2 Department of Cardiology, University Hospitals of North Midlands NHS Trust, Stoke-on-Trent ST4 6QG, UK; 3 Zeenat Qureshi Stroke Institute, Department of Neurology, University of Missouri, Columbia, MO 65212, USA; qureshai@gmail.com; 4 Liverpool Centre for Cardiovascular Science at University of Liverpool, Liverpool John Moores University & Liverpool Heart and Chest Hospital, Liverpool L69 7TX, UK; gregory.lip@liverpool.ac.uk; 5 Department of Clinical Medicine, Aalborg University, 9220 Aalborg, Denmark; 6 Department of Diabetes, University Hospital Birmingham, Birmingham B15 3TN, UK; 7 Division of Interventional Cardiology, Cardiovascular Diseases Department, University Hospital of Split (KBC Split), 21000 Split, Croatia

**Keywords:** type 1 diabetes mellitus, T1DM, cardiac disease, cerebrovascular disease, disease burden, mortality, inpatient, hospitalization cost

## Abstract

Background: This study aimed to evaluate the burden and impact of cardiac and cerebrovascular disease (CCD) on hospital inpatients with type 1 diabetes mellitus (T1DM). Methods: This is a retrospective nationwide cohort study of people with T1DM with or without CCD in the US National Inpatient Sample between 2016 and 2019. The in-hospital mortality rates, length of stay (LoS), and healthcare costs were determined. Results: A total of 59,860 T1DM patients had a primary diagnosis of CCD and 1,382,934 did not. The median LoS was longer for patients with CCD compared to no CCD (4.6 vs. 3 days). Patients with T1DM and CCD had greater in-hospital mortality compared to those without CCD (4.1% vs. 1.1%, *p* < 0.001). The estimated total care cost for all patients with T1DM with CCD was approximately USD 326 million. The adjusted odds of mortality compared to patients with non-CCD admission was greatest for intracranial hemorrhage (OR 17.37, 95%CI 12.68–23.79), pulmonary embolism (OR 4.39, 95%CI 2.70–7.13), endocarditis (OR 3.46, 95%CI 1.22–9.84), acute myocardial infarction (OR 2.31, 95%CI 1.92–2.77), and stroke (OR 1.47, 95%CI 1.04–2.09). Conclusions: The burden of CCD in patients with T1DM is substantial and significantly associated with increased hospital mortality and high healthcare expenditures.

## 1. Introduction

Type 1 diabetes mellitus (T1DM) is a chronic progressive lifelong condition associated with significant morbidity and increased mortality mainly from cardiovascular disease [1,2]. A large Swedish National Diabetes Registry showed that excess mortality was significant among patients with T1DM, and this was mainly due to cardiorenal complications, thus showing an unmet need in improvement in secondary prevention in this vulnerable patient strata [3]. Many adults with T1DM experience a low health-related quality of life, are more likely to be unemployed, and have more sickness days compared to the general population [4]. The morbidity from T1DM is substantial since it starts early in life and thus has a strong propensity for the development of microvascular and macrovascular complications [5,6]. Chronic hyperglycemia sustains and promotes oxidative stress, vascular inflammation, monocyte adhesion, and perturbations in the arterial wall and endothelium that leads to development of overt cardiovascular disease [7]. The T1DM accelerates atherosclerosis chiefly through the promotion of chronic and “low-grade” systemic inflammation and this contributes to the progression of valvular diseases and coronary artery disease [8,9]. These pathologic changes, in turn, can cause myocardial dysfunction and lead to heart failure with this risk being significantly greater in patients with T1DM due to the fact that disease duration is longer than in T2DM, which makes the likelihood of microvascular complications and detrimental effects of hyperglycemia greater [10]. It has been well established that diabetes inflicts adverse structural and metabolic changes of the myocardium with this entity being recognized as diabetic cardiomyopathy [11].

A meta-analysis by Cai et al. showed that the diagnosis of T1DM was strongly associated with an increased risk of several types of cardiovascular diseases including ischemic heart disease, myocardial infarction, heart failure, atrial fibrillation, and stroke [12]. Independent factors for ischemic stroke among patients with T1DM were the duration of diabetes, presence of diabetic nephropathy, higher hemoglobin A1c, higher systolic blood pressure, smoking, and degree of insulin resistance [13]. Interestingly, T1DM carries significantly greater risk of hemorrhagic stroke as well, compared to patients with type 2 diabetes mellitus, therefore highlighting general propensity of T1DM for potential cerebrovascular complications [14]. In line with this, the life expectancy in someone with T1DM is about 11 years shorter in men and 13 years in women, most of it being driven by a cardiovascular disease [15].

Taken together, there is a great clinical and public health interest in preventing and managing cardiovascular and cerebrovascular disease in patients with T1DM.

The burden and impact of cardiac and cerebrovascular disease (CCD) on patients who are admitted to a hospital with T1DM are largely unknown. The underlying pathology in these conditions may be related to diseases of the peripheral vasculature, coronary arteries, and myocardium; structural cardiac changes; or cardiac electrical activity, which contributes to inpatient hospital admission. How frequently these conditions account for hospitalizations among patients with T1DM and how they affect outcomes on a large nationwide scale are not known and have not been examined yet. Hence, the need to consider population-level data is required to capture the frequency of relevant clinical events, especially those that are less common.

In this comprehensive analysis, we examined a representative, large, nationwide database consisting of hospital records from the United States to evaluate the CCD burden among patients with T1DM and their respective outcomes. In-hospital mortality, length of stay, and cost were explored in detail across a range of CCD conditions.

## 2. Materials and Methods

This manuscript was prepared in accordance with the recommendations of the STROBE checklist [16]. Ethical approval was not required as we analyzed a non-identifiable public large-scale dataset. We analyzed nationally representative data in the United States from the National Inpatient Sample (NIS). The NIS is a database created by the Healthcare Cost and Utilization Project, which is the largest publicly available all-payer inpatient healthcare database in the United States, which can be utilized to provide national estimates of inpatient utilization, access, costs, quality, and outcomes [17].

A retrospective nationwide cohort study was undertaken of all hospital records in the United States with a discharge diagnosis of T1DM between 2016 and 2019. These years were chosen because the hospital admission information was in ICD-9 codes prior to 2016 and excluded years beyond 2019 to avoid a possible confounding effect of the COVID-19 pandemic. We excluded patients with age < 18 years and missing values for death and sex. From this group of patients, the first ICD-10 diagnostic code was used to define CCD as described in detail in Appendix A Table A1. CCD included angina pectoris, acute myocardial infarction, pulmonary embolism, acute pericarditis, aortic valve disorder, mitral valve disorder, right-sided heart valve disorder, endocarditis, myocarditis, cardiomyopathy, heart failure, atrial fibrillation or flutter, 2° or 3° atrioventricular block, intracranial hemorrhage, subarachnoid hemorrhage, and cerebral infarction. The discharge diagnosis codes, which were up to 40, were used to define coexisting illnesses, and demographic, hospital information and outcome data (in-hospital mortality, length of stay, and costs) were available in the NIS dataset.

### Statistical Methods

A statistical analysis was performed on Stata 13 (College Station, TX, USA). A *p*-value < 0.05 was considered as statistically significant. The hospital admissions were stratified by those who had CCD and those without CCD. Descriptive statistics were presented with the median and interquartile range (IQR) for continuous variables, and as a percent for categorical variables. The non-parametric equality-of-medians test on Stata was used to determine if there were any statistical differences for continuous variables and the Chi^2^ test was used for categorical variables. The frequency of the different individual diagnoses that composed CCD was determined together with the rate of mortality associated with each diagnosis. Both the median and mean length of stay for the individual diagnoses were presented. The total cost in USD was derived from the total charge multiplied by the charge-to-cost ratio and the average cost was used together with the frequency of the condition to estimate the total cost per year for the hospitalizations associated with CCD.

## 3. Results

**Figure 1:**
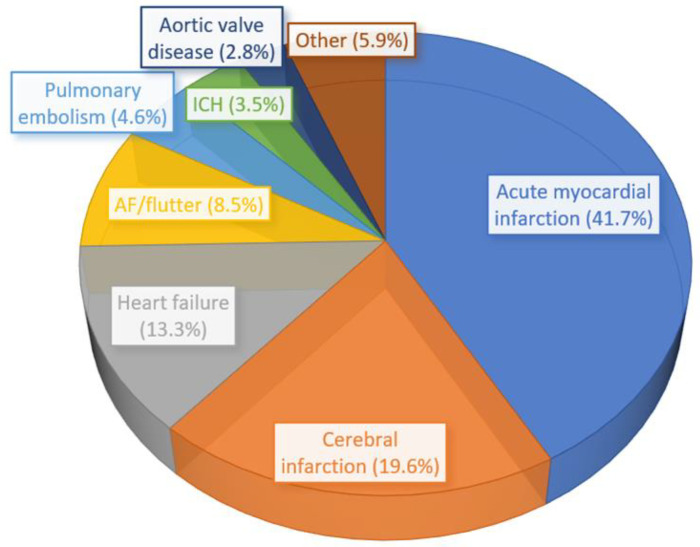
The distribution of the most common cardiovascular and cerebrovascular diseases in the cohort of 59,860 patients with T1DM.

**Figure A1:**
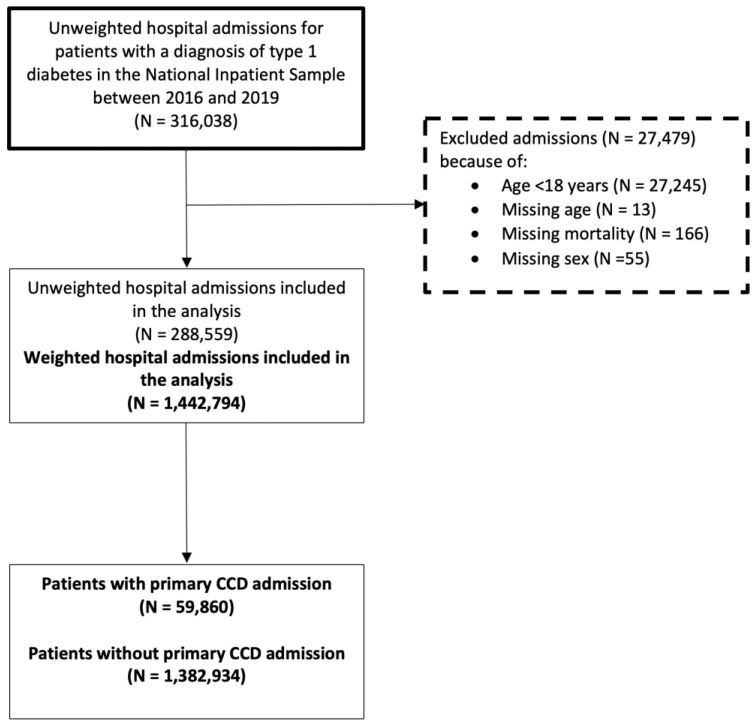
Patient inclusion flowchart.

There were a total of 1,442,794 weighted hospital admissions with T1DM included in the analysis (Figure A1, Appendix A). A total of 59,860 patients had a primary diagnosis of CCD and 1,382,934 did not have a primary diagnosis of CCD. The proportion of the different cardiovascular diagnoses that make up the CCD is shown in Figure 1. Acute myocardial infarction represented 41.7% of CCD followed by cerebral infarction (19.6%), heart failure (13.3%), and atrial fibrillation/atrial flutter (8.5%).

The demographics and comorbidities of included patients according to the presence or absence of a primary diagnosis of cardiovascular disease are shown in Table 1. The median age of patients with CCD was older compared to those without CCD (median of 60 vs. 39 years, *p* < 0.001). There were more female patients in the group without CCD compared to those with CCD (52.4% vs. 47.5%, *p* < 0.001). In terms of race, patients who were white had a greater proportion of patients with CCD compared to those without CCD (74.9% vs. 64.9%). The proportion of patients receiving Medicare was greater among patients who had CCD (55.4% vs. 32.7%) and the proportion of patients self-paying was greater for those without CCD (7.2% vs. 2.7%). The proportions of patients with obesity (18.2% vs. 10.1%, *p* < 0.001), hypertension (83.3% vs. 54.3%, *p* < 0.001), hyperlipidemia (64.5% vs. 31.7%, *p* < 0.001), previous myocardial infarction (16.4% vs. 5.8%, *p* < 0.001), previous stroke (16.2% vs. 6.7%, *p* < 0.001), previous heart failure (28.6% vs. 7.8%), and chronic lung disease (19.1% vs. 14.7%, *p* < 0001) were greater in the group of patients with T1DM and primary CCD compared to the group without CCD.

The mean and median length of hospital stay were significantly longer for patients with CCD (5.7 and 4 days vs. 4.6 and 3 days, respectively) compared to no CCD and the healthcare costs were significantly higher (mean of USD 21,802 and median of USD 13,762 vs. mean of USD 11,924 and median of USD 7113, respectively).

The in-hospital mortality rate was 4.1% for T1DM patients with CCD vs. 1.1% for T1DM patients without CCD (*p* < 0.001).

The length of hospital stay and cost of admissions for different primary diagnoses of CCD in patients with T1DM are shown in Table 2.

The median length of stay was longest for patients with right-sided heart valve disease, endocarditis, subarachnoid hemorrhage, mitral valve disease, and intracranial hemorrhage. The median cost was greatest for patients with right-sided heart valve disease followed by patients with mitral valve disease and those with aortic valve disease and subarachnoid hemorrhage. The estimated cost of hospitalizations for all admissions with a primary diagnosis of CCD was approximately USD 326 million each year with the admissions for acute myocardial infarction costing USD 163 million per year.

**Figure 2:**
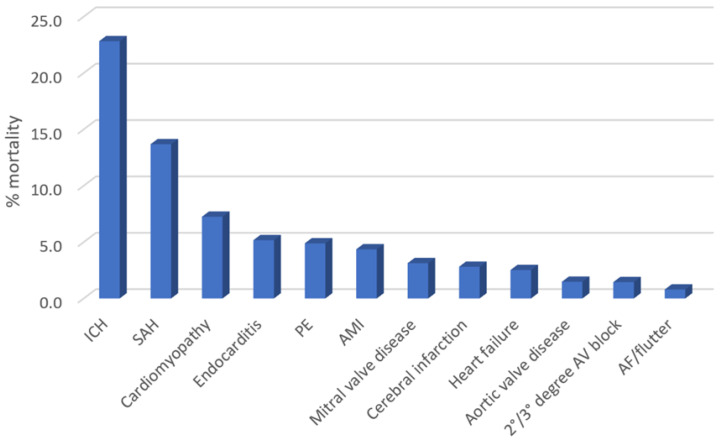
The in-hospital mortality of patients with T1DM stratified according to different primary and specific diagnoses of cardiovascular and cerebrovascular disease.

The rates of in-hospital mortality according to the different primary diagnoses of CCD are depicted in Figure 2. The mortality rate was greatest for patients admitted with intracranial hemorrhage (22.8%), subarachnoid hemorrhage (13.7%), cardiomyopathy (7.3%), endocarditis (5.2%), and pulmonary embolism (4.9%).

After adjustments for demographics and comorbidities, the multivariable-adjusted odds of in-hospital mortality compared to patients with non-CCD admission were greatest for intracranial hemorrhage (OR 17.37, 95%CI 12.68–23.79, *p* < 0.001), pulmonary embolism (OR 4.39, 95%CI 2.70–7.13, *p* < 0.001), endocarditis (OR 3.46, 95%CI 1.22–9.84, *p* = 0.020), acute myocardial infarction (OR 2.31, 95%CI 1.92–2.77, *p* < 0.001), and cerebral infarction (OR 1.47, 95%CI 1.04–2.09, *p* = 0.030) (Table 3). Finally, most common single primary diagnostic codes for patients that died were sepsis, T1DM with ketoacidosis without coma, non-ST elevation myocardial infarction, acute respiratory failure with hypoxia, and cardiac arrest with unspecified cause.

Finally, we provide an ancillary analysis of the impact of various common comorbidities on outcomes among patients that were hospitalized with T1DM. Table A2 (Appendix A) shows the impact of comorbidities (obesity, hypertension, hyperlipidemia, history of MI, stroke, HF, CKD, chronic lung disease, cancer, and dementia) on mortality, mean length of stay, and mean cost. The most common primary diagnoses for patients with T1DM admitted to a hospital due to non-CCD diagnoses were diabetic ketoacidosis without coma, sepsis, diabetic autonomic neuropathy, hyperglycemia, acute kidney injury, and hypoglycemia (Table A3, Appendix A).

## 4. Discussion

T1DM influences morbidity, length of stay, and mortality in patients with CCD. In a large, nationwide analysis of US data, we show that the CCD-related hospitalizations among patients with T1DM impose a great burden for hospital systems.

It has been well established that cardiovascular disease is the leading cause of death in patients with T1DM and while advancements have been made in management of microvascular complications, similar progress in reducing macrovascular complications remains a challenge [18]. In a study of 239 patients with T1DM who developed cardiovascular disease, microvascular disease including diabetic retinopathy, kidney disease, and cardiovascular autonomic neuropathy was associated with subsequent risk of major adverse cardiovascular events after adjusting for age and HbA1c [19]. Previously, the DCCT study showed that good glycemic control can reduce microvascular complications, but the effect on macrovascular complications is not clear [20]. Furthermore, data from the same study showed that variability in HbA1c is predictive of the development and progression of retinopathy and nephropathy in T1DM [21]. The microvascular complications of diabetes appear to cluster as one study of electronic medical records from a hospital in Denmark suggests that neuropathy and diabetic kidney disease often coexist as does retinopathy with both kidney disease and neuropathy [22]. Nevertheless, better management of cardiovascular risk factors and comorbidities in patients with T1DM, which includes adequate blood pressure control, lipid management, and lifestyle interventions, has reduced the burden of cardiovascular disease, but it is not clear what happens to patients with T1DM and underlying CCD when they are admitted to the hospital and what their outcomes are.

In our study, we analyzed the effect of CCD in patients with T1DM admitted to a nationwide array of US hospitals, along with analyzing individual CCD conditions that drive morbidity and mortality.

Many studies consider cardiovascular disease as an outcome, which refers primarily to ischemic heart disease and stroke with or without peripheral vascular disease. However, the vascular nature of the term cardiovascular disease precludes important cardiological conditions such as heart valve disease, arrhythmias, infective endocarditis, and inflammatory conditions of the heart muscle or pericardium and our study tried to account for these deficiencies in the literature.

There is strong evidence that T1DM will increase atherosclerotic risk factors, which can then independently increase cardiovascular risk, but whether either the diabetes itself or the associated risk factors change the risk for CCD is not known [23]. This is important as patients may develop a CCD condition because of the diabetes and risk factors or they may develop the condition independently, but their outcomes are affected by either the diabetes or risk factors. As Verges previously stated—cardiovascular risk remains high among well-controlled T1DM patients without traditional cardiovascular risk factors, thus suggesting other potential factors that drive poor outcomes [7]. Therefore, in the present analysis, it was important to perform multivariable adjustment for comorbid conditions and other factors, which may impact outcomes in a population of patients with T1DM.

The frequency of comorbid conditions and absolute mortality burden of CCD in type 1 diabetes should be considered. We show that mortality rates in patients with intracranial hemorrhage, subarachnoid hemorrhage, cardiomyopathy, and infective endocarditis are high. Several studies have evaluated these conditions in patients with T1DM. A case–control study of 120 patients with intracranial hemorrhage and 135 control patients with low back pain found that diabetes mellitus was more prevalent in the group with intracranial hemorrhage (33.1% vs. 22.2%), but it was not clear if there were any patients with T1DM [24]. A prospective cohort study of 4,083 patients with T1DM reported that during a median follow-up of 9.4 years, 15 patients developed subarachnoid hemorrhage and 4 had died [25]. For cardiomyopathy, a Swedish cohort study of 20,985 patients with T1DM found that there was a 4-fold increase in risk of development of heart failure for patients with HbA1c ≥10.5% compared to patients with HbA1c <6.5% [26]. Furthermore, a recent meta-analysis showed that patients with T1DM had 3-fold greater risk of developing heart failure, compared to controls without T1DM, and this was even more pronounced among women (had nearly 5-fold risk in that study) [27]. Regarding infective endocarditis, in a study of 559 patients with definite infective endocarditis, 13% of patients had T1DM and insulin-dependent diabetes was associated with a 4.7-fold increase in odds of in-hospital mortality [28].

According to results that we report, these conditions were not frequent population-wide and the estimated population deaths over the study years were 485 for intracranial hemorrhage and fewer than 100 deaths for the other three conditions. These low event rates even on a population level reflect the importance of national evaluations and a large nationwide analysis that can capture these nuances. While the mortality rate is lower, the estimated absolute number of deaths is greater for acute myocardial infarction (n = 1090). Overall, there were 17,420 deaths in the cohort and 14.1% (n = 2460) were due to CCD. The major non-CCD causes of mortality were sepsis, which caused over 4000 deaths, and ketoacidosis without coma, acute respiratory failure with hypoxia, and cardiac arrest, which were more than 500 deaths for each condition.

In our study, we show that patients with T1DM and CCD are different in terms of age and comorbidities compared to their counterparts without CCD. The comorbidities may be relevant because they may require management, which contributes to prolonged hospital stay and cost. In addition, an important consideration is whether these factors impact clinical decision making as the elderly and presences of coexisting illness can affect risk of undertaking procedures. The broad nature of the conditions captured in CCD is important; there may be interventional and surgical options for management in some of the conditions such as infective endocarditis, valvular heart disease, and coronary artery disease. While attempts were made with adjustments in our analysis, the likely reality is that we are not able to account for the entire effect of these factors.

Finally, all patients should also be under the care of a diabetes specialist team and there should be locally established care pathways with shared care where appropriate. These pathways may help reduce any missed opportunities to prevent the progression of diabetes and complications and enable addressing early detection problems when they develop.

### Limitations

This analysis has several limitations. First, we used the primary ICD-10 codes as the method of determining the primary reason for admission. It is possible that there may be more than one key condition that resulted in the condition. Second, the NIS does not contain patient-level identifiers so patients who survive admission may be readmitted and considered within a given year and across different years. Third, we do not have information about the management of patients, which influences mortality, length of stay, and cost.

## 5. Conclusions

Approximately 4% of admissions for patients with a primary diagnosis of T1DM are for CCD. These admissions to a hospital are important because they are associated with a four-fold increase in mortality, longer mean length of stay, and double the mean cost of admission compared to admissions without a primary diagnosis of CCD. In particular, the cost of admissions with heart valve disease is high, which is greater than USD 40,000. The mortality rate is more than 5% for patients admitted with intracranial hemorrhage, subarachnoid hemorrhage, and cardiomyopathy. These findings suggest that the CCD in patients with T1DM is associated with mortality and is a burden to hospital services so measures should be taken to manage cardiovascular risk factors to prevent onset of CCD in patients with T1DM.

## Figures and Tables

**Table 1:** Demographics of patients with T1DM who were admitted to hospital stratified according to the primary diagnosis of cardiac and cerebrovascular disease (CCD).

Variable	Total (*n* = 1,442,794)	CCD (*n* = 59,860)	No CCD (*n* = 1,382,934)	*p*-Value
Median age [IQR]	40 [28 to 56]	60 [48 to 70]	39 [28 to 55]	<0.001
Female	52.2%	47.5%	52.4%	<0.001
Race				<0.001
White	65.0%	74.9%	64.6%	
Black	20.5%	13.5%	20.8%	
Hispanic	10.2%	7.1%	10.3%	
Asian or Pacific Islander	1.2%	1.5%	1.2%	
Native American	0.8%	0.6%	0.8%	
Other	2.3%	2.4%	2.3%	
Smoking	1.6%	1.1%	1.6%	<0.001
Alcohol misuse	2.3%	1.4%	2.3%	<0.001
Elective	9.6%	7.8%	9.7%	<0.001
Weekend admission	24.0%	23.2%	24.0%	0.047
Season of admission				<0.001
Spring	25.0%	26.2%	24.9%	
Summer	24.8%	25.1%	24.8%	
Fall	25.1%	23.5%	25.2%	
Winter	25.2%	25.2%	25.1%	
Year				<0.001
2016	24.2%	29.6%	24.0%	
2017	24.9%	23.7%	25.0%	
2018	25.3%	23.2%	25.4%	
2019	25.5%	23.5%	25.6%	
Hospital bed size				<0.001
Small	20.2%	17.0%	20.4%	
Medium	28.3%	27.6%	28.3%	
Large	51.5%	55.5%	51.3%	
Primary expected payer				<0.001
Medicare	33.7%	55.4%	32.7%	
Medicaid	29.3%	14.%	30.%	
Private insurance	25.9%	25.5%	27.%	
Self-pay	7.%	2.7%	7.2%	
No charge	0.5%	0.2%	0.5%	
Other	2.6%	2.3%	2.6%	
ZIP income quartile				<0.001
1st–25th	33.7%	28.9%	33.9%	
26th–50th	27.3%	26.8%	27.3%	
51st–75th	23.0%	24.9%	22.9%	
76th–100th	16.0%	19.4%	15.9%	
Obesity	10.5%	18.2%	10.1%	<0.001
Hypertension	55.5%	83.3%	54.3%	<0.001
Hyperlipidemia	33.1%	64.5%	31.7%	<0.001
Previous myocardial infarction	6.2%	16.4%	5.8%	<0.001
Previous stroke	7.1%	16.2%	6.7%	<0.001
Previous heart failure	8.6%	28.6%	7.8%	<0.001
Chronic kidney disease	62.5%	74.3%	61.8%	<0.001
Chronic lung disease	14.9%	19.1%	14.7%	<0.001
Cancer	3.2%	3.7%	3.2%	0.002
Dementia	2.4%	4.4%	2.4%	<0.001
Mean length of stay (SD)	4.7 ± 6.4	5.7 ± 7.0	4.6 ± 6.3	<0.001
Median length of stay [IQR]	3 [2 to 5]	4 [2 to 7]	3 [2 to 5]	<0.001
Mean cost (SD)	USD 12,333 ± 19,111	USD 21,802 ± 26,970	USD 11,924 ± 18,588	<0.001
Median cost [IQR]	USD 7271 [4478 to 13,183]	USD 13,762 [7551 to 26,071]	USD 7113 [4415 to 12,737]	<0.001
In-hospital mortality	1.2%	4.1%	1.1%	<0.001

CCD = cardiac and cerebrovascular disease, IQR = interquartile range, SD = standard deviation.

**Table 2:** Length of hospital stay and cost according to primary diagnosis of cardiac and cerebrovascular disease in patients with T1DM.

Primary CCD Diagnosis	Median Length of Stay [IQR]	Mean Length of Stay (SD)	Median Cost [IQR]	Mean Cost (SD)
Right-sided heart valve disease	12 [7 to 16]	12.0 ± 5.7	USD 54,433 [20,422 to 118,324]	69,373 ± 67,825
Endocarditis	9 [6 to 15]	13.7 ± 14.3	USD 22,182 [12,546 to 51,258]	36,825 ± 38,876
Subarachnoid hemorrhage	9 [5 to 16]	11.4 ± 10.0	USD 36,428 [23,138 to 65,209]	53,927 ± 54,180
Mitral valve disease	8 [4 to 11]	9.9 ± 9.4	USD 50,207 [33,881 to 71,083]	62,531 ± 54,586
Intracranial hemorrhage	5 [3 to 9]	8.5 ± 10.7	USD 16,132 [9313 to 30,006]	28,669 ± 36,988
Aortic valve disease	5 [2 to 8]	6.4 ± 6.5	USD 44,320 [32,266 to 58,626]	48,649 ± 32,260
Pulmonary embolism	4 [2 to 7]	5.9 ± 13.7	USD 9,521 [5,947 to 16,286]	16,172 ± 26,635
Acute myocardial infarction	4 [2 to 7]	5.7 ± 6.0	USD 18,893 [11,103 to 31,815]	26,128 ± 26,329
Heart failure	4 [2 to 7]	5.7 ± 5.4	USD 8857 [5534 to 14,987]	13,866 ± 20,559
Cardiomyopathy	3 [2 to 7]	6.6 ± 15.2	USD 12,842 [9101 to 30,014]	37,515 ± 89,745
Cerebral infarction	3 [2 to 6]	5.3 ± 6.6	USD 10,453 [6815 to 17,214]	15,701 ± 19,648
Acute pericarditis	3 [2 to 5]	4.5 ± 4.7	USD 8370 [5382 to 14,856]	11,972 ± 9917
2°/3° AV block	3 [2 to 5]	4.4 ± 4.1	USD 18,398 [12,151 to 28,144]	21,758 ± 15,220
Atrial fibrillation/flutter	3 [1 to 4]	3.3 ± 3.1	USD 7061 [4368 to 13,564]	11,623 ± 12,408
Myocarditis	3 [1 to 4]	2.7 ± 1.6	USD 15,778 [8954 to 19,741]	14,778 ± 6901
Angina	2 [1 to 3]	2.1 ± 1.5	USD 7594 [4694 to 10,125]	9209 ± 7220

CCD = cardiac and cerebrovascular disease, IQR = interquartile range, SD = standard deviation.

**Table 3:** Multivariable-adjusted odds of in-hospital mortality among patients with T1DM and primary CCD diagnosis compared in relation to patients with T1DM and no CCD diagnosis (used as a reference group).

Primary Diagnosis of CCD	Odds Ratio (95%CI) *	*p*-Value
Intracranial hemorrhage	17.37 (12.68–23.79)	<0.001
Pulmonary embolism	4.39 (2.70–7.13)	<0.001
Endocarditis	3.46 (1.22–9.84)	0.020
Acute myocardial infarction	2.31 (1.92–2.77)	<0.001
Cerebral infarction	1.47 (1.04–2.09)	0.030
Aortic valve disease	0.95 (0.35–2.60)	0.920
Heart failure	0.86 (0.60–1.24)	0.422
2°/3° AV block	0.74 (0.23–2.36)	0.627
Atrial fibrillation/flutter	0.43 (0.19–0.97)	0.043

* Adjusted for all variables in Table 1 except for length of stay and cost. CCD = cardiac and cerebrovascular disease, CI = confidence interval.

## Data Availability

The data used in this analysis may be purchased from the Healthcare Cost and Utilization Project (HCUP) website. The authors do not have permission to share the data used for the analysis.

## Appendix A

**Table A1:** ICD-10 codes for data analysis and their source.

Variable	Source	ICD-10 Code
Type 1 diabetes	I10_DX1/40	E10*
Angina pectoris	I10_DX1	I20*
Acute myocardial infarction	I10_DX1	I21*
Pulmonary embolism	I10_DX1	I26*
Acute pericarditis	I10_DX1	I30*
Aortic valve disorder	I10_DX1	I35*
Mitral valve disorder	I10_DX1	I34*
Right-sided heart valve disorder	I10_DX1	I36*, I37*
Endocarditis	I10_DX1	I33*, I38*, I39*
Myocarditis	I10_DX1	I40*, I41*
Cardiomyopathy	I10_DX1	I42*, I43*
Heart failure	I10_DX1	I50*
Atrial fibrillation/atrial flutter	I10_DX1	I48*
2°/3° AV block	I10_DX1	I44.1, I44.2
Intracranial hemorrhage	I10_DX1	I61*, I62*
Subarachnoid hemorrhage	I10_DX1	I60*
Cerebral infarction	I10_DX1	I63*
Smoking (tobacco use)	I10_DX1/40	Z72.0
Alcohol misuse	I10_DX1/40	F10.1
Obesity	I10_DX1/40	E66.0, E66.1, E66.2, E66.8, E66.9
Hypertension	I10_DX1/40	I10*, I11*, I12*, I13*, I15*, I16*
Hyperlipidemia	I10_DX1/40	E78.0*, E78.1, E78.2, E78.3, E78.4*, E78.5
Previous myocardial infarction	I10_DX1/40	I25.2
Previous stroke	I10_DX1/40	Z86.73, I69*
Previous heart failure	I10_DX1/40	I50.22, I50.23, I50.32, I50.33, I50.42, I50.43, I50.812, I50.813
Chronic kidney disease	I10_DX1/40	N18*
Chronic lung disease	I10_DX1/40	J40*–J47*
Any cancer	I10_DX1/40	C00*–C96*
Dementia	I10_DX1/40	F01*, F02*, F03*
Age	NIS Core	-
Sex	NIS Core	-
Month of admission	NIS Core	-
Weekend admission	NIS Core	-
Discharge weight	NIS Core	-
Discharge disposition	NIS Core	-
Elective admission	NIS Core	-
Length of stay	NIS Core	-
Primary expected payer	NIS Core	-
Race	NIS Core	-
Year	NIS Core	-
ZIP income quartile	NIS Core	-
Death	NIS Core	-
Total charge	NIS Core	-
Hospital bed size	NIS Hospital	-

* denotes all the sub-codes from the root code.

**Table A2:** Impact of comorbidities on outcomes in patients hospitalized with T1DM.

Comorbidity	Mortality with Comorbidity vs. without	*p*-Value	Mean Length of Stay with Comorbidity vs. without (days)	*p*-Value	Mean Cost with Comorbidity vs. without (USD)	*p*-Value
Obesity	1.2% vs. 1.2%	1.00	5.4 ± 6.6 vs. 4.6 ± 6.3	<0.001	14,970 ± 30,017 vs. 12,024 ± 19,076	<0.001
Hypertension	1.6% vs. 0.7%	<0.001	5.3 ± 6.7 vs. 3.9 ± 5.7	<0.001	14,435 ± 20,165 vs. 9712 ± 17,355	<0.001
Hyperlipidemia	1.4% vs. 1.1%	<0.001	5.1 ± 6.0 vs. 4.5 ± 6.5	<0.001	14,308 ± 19,205 vs. 11,358 ± 18,988	<0.001
History of myocardial infarction	2.0% vs. 1.2%	<0.001	5.2 ± 5.6 vs. 4.6 ± 6.4	<0.001	14,814 ± 17,841 vs. 12,169 ± 19,180	<0.001
History of stroke	2.0% vs. 1.2%	<0.001	5.7 ± 7.1 vs. 4.6 ± 6.3	<0.001	14,377 ± 17,330 vs. 12,176 ± 19,232	<0.001
History of heart failure	3.4% vs. 1.0%	<0.001	6.8 ± 7.9 vs. 4.5 ± 6.1	<0.001	18,773 ± 25,933 vs. 11,726 ± 18,221	<0.001
Chronic kidney disease	2.2% vs. 0.8%	<0.001	6.0 ± 7.5 vs. 4.1 ± 5.8	<0.001	16,539 ± 23,959 vs. 10,621 ± 16,436	<0.001
Chronic lung disease	1.5% vs. 1.2%	<0.001	5.1 ± 6.5 vs. 4.6 ± 6.3	<0.001	13,329 ± 18,195 vs. 12,159 ± 19,621	<0.001
Cancer	4.4% vs. 1.1%	<0.001	6.9 ± 8.5 vs. 4.6 ± 6.3	<0.001	20,823 ± 29,978 vs. 12,051 ± 18,575	<0.001
Dementia	3.6% vs. 1.2%	<0.001	6.9 ± 10.4 vs. 4.6 ± 6.2	<0.001	14,709 ± 20,252 vs. 12,274 ± 19,078	<0.001

**Table A3:** The most common primary diagnosis among patients with T1DM and non-CCD-related hospital admissions.

Primary Diagnosis Code for Non-CCD Admission	Number of Admissions
Diabetic ketoacidosis without coma	453,550
Sepsis	78,135
Diabetic autonomic polyneuropathy	43,080
Hyperglycemia	39,435
Acute kidney injury	25,620
Hypoglycemia	22,930
Other complication of type 1 diabetes	16,200
Foot ulcer	13,190
Pre-existing type 1 diabetes in childbirth	12,225
Pneumonia	12,170
Diabetic peripheral angiopathy and gangrene	8380
Urinary tract infection	7615
Acute pancreatitis without necrosis or infection	6975
Mechanical complication of insulin pump	6880
Hyperkalemia	6310
Diabetic ketoacidosis with coma	6245
Diabetic chronic kidney disease	5970
Sepsis due to *E. coli*	5845
Major depressive disorder, recurrent severe without psychotic features	5790
Acute respiratory failure with hypoxia	5720
